# Fibrates and the risk of cardiovascular outcomes in chronic kidney disease patients

**DOI:** 10.1093/ndt/gfad248

**Published:** 2023-11-27

**Authors:** Hirohito Goto, Ken Iseri, Noriko Hida

**Affiliations:** Center for Novel and Exploratory Clinical Trials (Y-NEXT), Yokohama City University Hospital, Kanagawa, Japan; Department of Clinical Pharmacy, Division of Clinical Research and Development, School of Pharmacy, Showa University, Tokyo, Japan; Department of Clinical Pharmacy, Division of Clinical Research and Development, School of Pharmacy, Showa University, Tokyo, Japan; Department of Clinical Pharmacy, Division of Clinical Research and Development, School of Pharmacy, Showa University, Tokyo, Japan

**Keywords:** cardiovascular events, chronic kidney disease, dyslipidemia, fibrates, triglycerides

## Abstract

**Background:**

The high risk of major adverse cardiovascular events (MACE) in patients with chronic kidney disease (CKD) has been well described. However, the efficacy of fibrates on the risk of MACE in patients with CKD remains unclear.

**Methods:**

We conducted a nested case–control study using data from a large administrative database that included more than 1.5 million Japanese patients. We defined cases as CKD patients with incidences of MACE and matched them with controls based on age, sex, calendar year of cohort entry and CKD stage. Fibrate exposure timing was categorized as current, recent or past. A conditional logistic regression analysis was used to investigate the association between fibrate use and the risk of MACE.

**Results:**

Our study included 47 490 patients with CKD, with 15 830 MACE identified during a median follow-up of 9.4 months. The numbers of fibrates used during the study period were 556 (3.5%) in the case group and 1109 (3.5%) in the control group. Fibrate use was significantly associated with a decreased risk of MACE [odds ratio (OR) 0.84; 95% confidence interval (CI) 0.75–0.94], particularly for current (OR 0.81; 95% CI 0.68–0.97) and recent use (OR 0.65; 95% CI 0.48–0.90). Regarding the class effect of fibrates, pemafibrate use, but not bezafibrate or fenofibrate use, was significantly associated with a decreased risk of MACE (OR 0.73; 95% CI 0.528–0.997).

**Conclusion:**

Recent and current fibrate use, especially pemafibrate use, was associated with a reduced risk of MACE in patients with CKD. This suggests the potential benefits of continuous fibrate therapy and the possible superiority of pemafibrate over other fibrates. However, further investigations in different populations are required to confirm the generalizability of these findings.

## Graphical Abstract

**Graphical Abstract:**
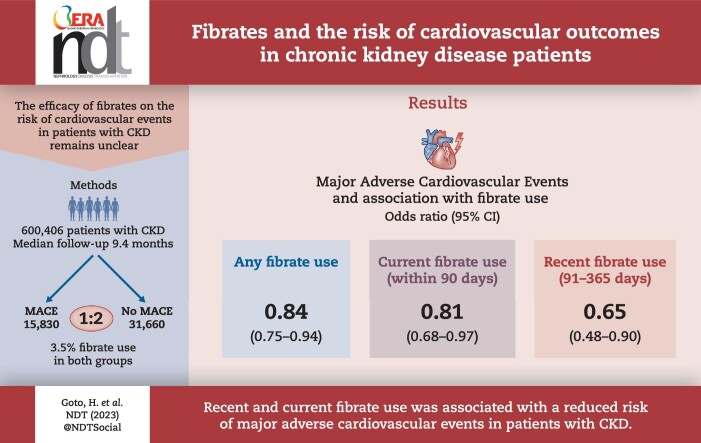

KEY LEARNING POINTS **What was known:**

- Patients with chronic kidney disease (CKD) have a higher risk of major adverse cardiovascular events (MACE) than the general population.
- Information regarding the association between fibrate use and the risk of MACE in patients with CKD is limited.
- Furthermore, the class effect of fibrates on MACE is not fully understood.

**This study adds:**

- Recent and current use of fibrates, but not past use, was significantly associated with reduced risks of MACE in patients with CKD.
- Among the classes of fibrates, a significant association was observed between current pemafibrate use, but not bezafibrate and fenofibrate use, and a reduced risk of MACE.

**Potential impact:**

- The optimal use of fibrates in patients with CKD may lead to better clinical outcomes.
- Among fibrates, pemafibrate may be a better choice than other fibrates.

## INTRODUCTION

Declining kidney function results in various complications, such as vascular calcification and atherosclerosis, as the kidney plays a crucial role in the management of systemic mineral metabolism through the direct and indirect production of various hormones [1]. The development of atherosclerosis can lead to an increase in cardiovascular events, which remain the leading causes of morbidity and mortality in patients with chronic kidney disease (CKD) [1]. The risk of developing cardiovascular events in patients with CKD is 1.3–4 times higher than that in the general population [2, 3]. The prevention of cardiovascular events is important for improving the prognosis of patients with CKD.

Managing lipid levels, including the level of triglycerides, is beneficial in reducing cardiovascular events in patients with atherogenic dyslipidemia. Various national and international guidelines for lipid management have set triglyceride control targets, and the use of fibrates is recommended in patients with high levels of triglycerides [4–6]. Fibrates are effective in reducing triglyceride levels and increasing high-density lipoprotein cholesterol levels [7]. However, as most fibrates are excreted renally, its use in CKD patients receiving statins was contraindicated until 2018 due to a potentially increased risk of rhabdomyolysis. However, considering its beneficial effects in combination therapy, this contraindication was lifted in Japan in 2018, even for patients with CKD [8]. The prescription of fibrates in Japan may increase following the lifting of contraindications for the combined use of fibrates and statins.

Gemfibrozil use is significantly associated with a reduced incidence of major adverse cardiovascular events (MACE) in the general population and patients with type 2 diabetes [9, 10]. A population-based study in Taiwan showed that fibrates reduced the risk of cardiovascular events and delayed dialysis initiation [11]. However, the data are limited and inconsistent in the CKD population [12]. Fibrate use is poorly studied in the Japanese population, especially in patients with CKD, as its use was limited in the past because of the contraindication for combined use with statins. Furthermore, information regarding the class effect of fibrates on the risk of MACE is sparse [13].

This study aimed to investigate the association between fibrate use and the risk of MACE in patients with CKD using a large national database.

## MATERIALS AND METHODS

### Patients and data sources

Medical Data Vision Co., Ltd (MDV) maintains one of the largest hospital claims registries in Japan, which contains records of individual prescriptions, procedures, examinations, surgeries, hospitalizations and clinical diagnoses based on the International Classification of Disease 10th Revision (ICD-10) codes from more than 460 hospitals. This database includes data from more than 40 million people, accounting for 31% of Japan's total population (https://en.mdv.co.jp/). As the data used in this study were already anonymized, institutional review board approval and patient consent were not required in accordance with Japanese ethical guidelines [14]; the utilization of de-identified data complied with local regulations.

**Figure 1:**
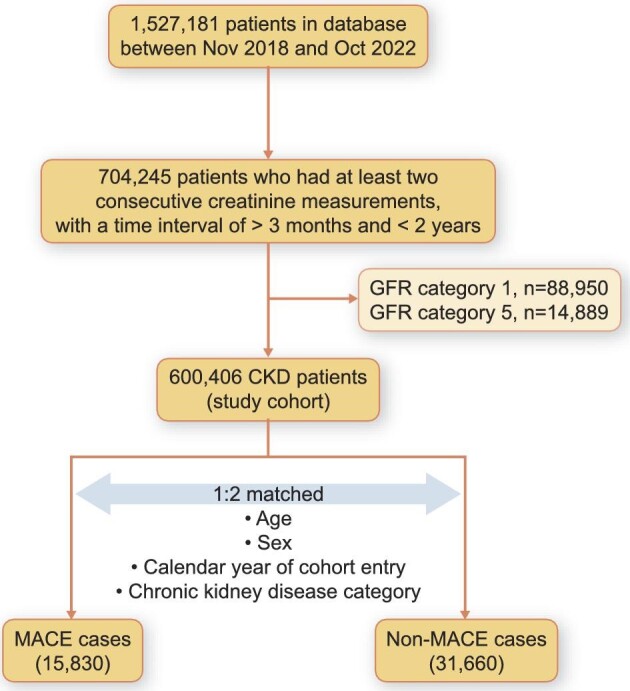
Flow chart of the study.

For our nested case–control study, we extracted data from the MDV database for 1 527 181 patients (aged ≥18 years) who had undergone plasma or serum creatinine measurement at least once between 1 November 2018 and 31 October 2022. We defined CKD as having at least two consecutive creatinine measurements, with a time interval of >3 months and <2 years, indicating estimated glomerular filtration rates (eGFR) ≥15 mL/min/1.73 m^2^ and <90 mL/min/1.73 m^2^. The date of the second measurement meeting this criterion was considered the cohort entry date (Fig. 1).

### Cases and controls

Cases were defined based on the incidence of MACE, which are a composite of myocardial infarction, stroke, heart failure and death due to cardiovascular diseases (Supplementary data, Table S1). The diagnostic accuracy of the ICD-10 records in this database has been validated [15]. The index date was the first date of a MACE. The cases and controls were matched in a 1:2 ratio based on age, sex, calendar year of cohort entry and CKD stage at cohort entry. To ensure a comparable follow-up period between the cases and controls, each matched control was assigned the same index date as their corresponding case and met the criteria of being alive and not having experienced MACE. Using this approach, the risk set for a particular case comprised all at-risk individuals. Under this definition, another case could be considered a potential control if it developed MACE at a later date.

### Fibrate exposure

The cases and controls were assigned to two distinct and mutually exclusive groups based on their exposure to fibrates (clofibrate, clinofibrate, bezafibrate, fenofibrate and pemafibrate) before the index date, as assessed using Anatomical Therapeutic Chemical Classification (ATC) codes (Supplementary data, Table S2). We determined the timing of fibrate use prior to the index date by analyzing the last date of the dispensed drug. Furthermore, we classified fibrate use into three distinct groups based on the timing with regard to the index date: (i) current use (within 90 days before the index date), (ii) recent use (between 91 and 365 days before the index date) and (iii) past use (more than 366 days before the index date).

### Study covariates

For all cases and controls, study covariates included comorbid conditions and medications. Comorbidities within 1 year before the index date, including diabetes, atrial fibrillation/flutter, ischemic heart disease, cerebrovascular disease, peripheral vascular disease, and chronic pulmonary disease, were assessed using ICD-10 codes (Supplementary data, Table S1). Medications included angiotensin-converting–enzyme inhibitors/angiotensin-receptor blockers, β-blockers, calcium channel blockers, statins, diuretics, anticoagulants, antiplatelet agents, sodium-glucose cotransporter 2 inhibitor, glucagon-like peptide-1 receptor agonist, glucocorticoid inhalant, steroids, non-steroidal anti-inflammatory drugs (NSAIDs), opioids and antidepressants, which were assessed using ATC codes (Supplementary data, Table S2).

### Statistical analysis

Data are expressed as median (25th–75th percentile), percentage or odds ratio (OR), as appropriate. Comparisons between the two groups were performed using the non-parametric Wilcoxon test for continuous variables and the chi-squared test for nominal variables. To explore the correlation between the use of fibrates and the risk of MACE, we employed conditional logistic regression, which allowed us to calculate the ORs for MACE. The time-matched nested case–control methodology adopted in this study delivers unbiased estimations of the rate ratio and 95% confidence intervals (CIs); all previously mentioned covariates were adjusted as clinically relevant confounding variables. Statistical significance was set at *P* < .05. Statistical analyses were performed using Stata 16.0 (Stata Corporation, College Station, TX, USA).

## RESULTS

### Baseline characteristics

**Figure 2:**
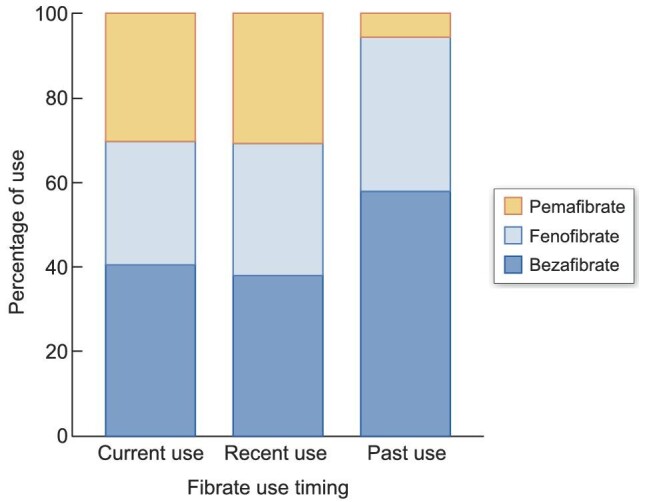
Stacked bar chart showing the percentage of bezafibrate, fenofibrate and pemafibrate use at each prescription timepoint.

In our cohort of 47 490 patients with CKD, we identified 15 830 cases of MACE during a median follow-up of 9.4 months (interquartile range 3.5–19.1). Table 1 shows the demographic and clinical characteristics of the patients, and their matched controls at the index date. The median patient age was 81 years, and 57% of the patients were male. The frequencies of comorbidities and co-medications (except opioids) listed in Table 1 were significantly higher for cases than for controls. The types of last-prescribed fibrates before the index data were comparable between cases and controls. Bezafibrate (48%) was the most used fibrate. When sorting as per the timing of prescriptions, pemafibrate was used less frequently in the past use than in recent and current use times (Fig. 2).

**Table 1:** Baseline characteristics of the study participants.

Characteristic	Cases	Controls	*P*-value
Sample size	15 830	31 660	
Sex, *n* (%)			
Women	6794 (42.9)	13 588 (42.9)	
Age	81 (74–87)	81 (74–87)	1.00
Age categories, *n* (%)			1.00
18–49 years	232 (1.5)	464 (1.5)	
50–59 years	554 (3.5)	1108 (3.5)	
60–69 years	1615 (10.2)	3230 (10.2)	
70–79 years	4663 (29.5)	9326 (29.5)	
80–89 years	6407 (40.5)	12 814 (40.5)	
≥90 years	2359 (14.9)	4718 (14.9)	
eGFR	50 (36, 64)	50 (36, 64)	.79
GFR categories, *n* (%)			.86
Category 2	5242 (33.1)	10 505 (33.2)	
Category 3a	4528 (28.6)	8976 (28.4)	
Category 3b	3801 (24.0)	7698 (24.3)	
Category 4	2259 (14.3)	4481 (14.2)	
Comorbid conditions, *n* (%)			
Diabetes	6797 (42.9)	9956 (31.4)	<.001
Atrial fibrillation/flutter	2924 (18.5)	1823 (5.8)	<.001
Ischemic heart disease	3015 (19.0)	2676 (8.5)	<.001
Cerebrovascular disease	3373 (21.3)	3695 (11.7)	<.001
Peripheral vascular disease	1810 (11.4)	2271 (7.2)	<.001
Chronic pulmonary disease	2159 (13.6)	2521 (8.0)	<.001
Medications, *n* (%)			
Fibrate	556 (3.5)	1109 (3.5)	.93
Bezafibrate	273 (1.7)	521 (1.6)	
Fenofibrate	171 (1.1)	373 (1.2)	
Pemafibrate	112 (0.7)	215 (0.7)	
ACEi/ARB	9786 (61.8)	14 832 (46.8)	<.001
β-blocker	7521 (47.5)	8177 (25.8)	<.001
CCB	9732 (61.5)	16 449 (52.0)	<.001
Statins	6225 (39.3)	10 674 (33.7)	<.001
Diuretics	9430 (59.6)	10 955 (34.6)	<.001
Anticoagulants warfarin	2767 (17.5)	2438 (7.7)	<.001
Anticoagulants DOAC	4909 (31.0)	4819 (15.2)	<.001
Antiplatelet agents	7521 (47.5)	10 149 (32.1)	<.001
SGLT2	1654 (10.4)	1699 (5.4)	<.001
GLP1	350 (2.2)	439 (1.4)	<.001
Glucocorticoid inhalant	1539 (9.7)	2450 (7.7)	<.001
Steroids	2362 (14.9)	4436 (14.0)	<.001
Nsaids	7863 (49.7)	15 523 (49.0)	<.001
Opioids	1181 (7.5)	3205 (10.1)	<.001
Antidepressant	1890 (11.9)	2961 (9.4)	<.001
Calendar year of cohort entry, *n* (%)			1.00
2019	8362 (52.8)	16 724 (52.8)	
2020	3087 (19.5)	6174 (19.5)	
2021	3481 (22.0)	6962 (22.0)	
2022	900 (5.7)	1800 (5.7)	

Values are mean (standard deviation), median (interquartile range) or *n* (%).

ACEi/ARB, angiotensin-converting enzyme inhibitors/angiotensin-receptor blockers; CCB, calcium-channel blockers; SGLT2, sodium-glucose cotransporter 2 inhibitor; GLP1, glucagon-like peptide-1 receptor agonist

### Effect of fibrate use on the MACE risk

The frequencies of fibrate use during the study period were similar between the two groups: 556 cases (3.5%) and 1109 controls (3.5%). A similar tendency was observed for the differences in the frequencies of fibrate use between the two groups according to prescription timing.

Based on the conditional logistic regression analysis after adjusting for potential confounders, fibrate use during the study period was significantly associated with a decreased risk of MACE (OR 0.84; 95% CI 0.75–0.94). Similarly, significant associations were observed between recent and current use and the risk of MACE (current use: OR 0.81; 95% CI 0.68–0.97, recent use: OR 0.65; 95% CI 0.48–0.90). However, past use was not significantly associated with the risk of MACE (OR 0.94; 95% CI 0.79–1.12)(Table 2).

**Table 2:** Multivariate adjusted ORs for the risk of MACE.

	*n* (%)		OR (95% CI)	
	Cases	Controls	Adjusted	*P*-value
No fibrate use	15 274 (96.5)	30 551 (96.5)	1 (reference)	
Fibrate use	556 (3.5)	1109 (3.5)	0.84 (0.75–0.94)	<.05
Current use (within 90 days)	225 (1.4)	486 (1.5)	0.81 (0.68–0.97)	<.05
Recent use (91–365 days)	71 (0.5)	156 (0.5)	0.65 (0.48–0.90)	<.05
Past use (over 365 days)	260 (1.6)	467 (1.5)	0.94 (0.79–1.12)	.49

Covariates list: age, sex, eGFR category, diabetes, atrial fibrillation/flutter, ischemic heart disease, cerebrovascular disease, peripheral vascular disease, chronic pulmonary disease, angiotensin-converting enzyme inhibitors/angiotensin-receptor blockers, β-blockers, calcium channel blockers, statins, diuretics, anticoagulants, antiplatelet agents, sodium-glucose cotransporter 2 inhibitor, glucagon-like peptide-1 receptor agonist, glucocorticoid inhalant, steroids, NSAIDs, opioids, antidepressant and calendar year of cohort entry.

### The class effect of fibrate on the risk of MACE

As we observed significant associations between current and recent use and the risk of MACE, we investigated the class effect of fibrates on the risk of MACE. The frequency of current no-fibrate use was similar between the two groups. Based on a multivariate conditional logistic regression analysis, current pemafibrate use was significantly associated with a decreased risk of MACE (OR 0.73; 95% CI 0.528–0.997) compared with non-current use. However, there was no significant association between current bezafibrate and fenofibrate use and the risk of MACE (Table 3).

**Table 3:** Multivariate adjusted ORs for the risk of MACE in relation to the current use of bezafibrate, fenofibrate and pemafibrate compared with non-use.

	*n* (%)		OR (95% CI)	
	Cases	Controls	Adjusted	*P*-value
No current fibrate use	15 605 (98.6)	31 174 (98.5)	1 (reference)	
Current use of bezafibrate	92 (0.6)	195 (0.6)	0.91 (0.692–1.201)	.509
Current use of fenofibrate	61 (0.4)	147 (0.5)	0.77 (0.554–1.082)	.134
Current use of pemafibrate	72 (0.5)	144 (0.5)	0.73 (0.528–0.997)	<.05

Covariates list: age, sex, eGFR category, diabetes, atrial fibrillation/flutter, ischemic heart disease, cerebrovascular disease, peripheral vascular disease, chronic pulmonary disease, angiotensin-converting enzyme inhibitors/angiotensin-receptor blockers, β-blockers, calcium channel blockers, statins, diuretics, anticoagulants, antiplatelet agents, sodium-glucose cotransporter 2 inhibitor, glucagon-like peptide-1 receptor agonist, glucocorticoid inhalant, steroids, NSAIDs, opioids, antidepressant and calendar year of cohort entry.

## DISCUSSION

Our nested case–control study showed a significant association between recent and current fibrate use and a reduced risk of MACE in patients with CKD. However, this association was not observed in patients treated with fibrates in the past. Regarding the class effect of fibrates on the risk of MACE, only the current use of pemafibrate was significantly associated with a reduced risk of MACE. There were no significant associations between the current use of bezafibrate and fenofibrate and the risks of MACE.

A previous meta-analysis showed that fibrate use was associated with lower cardiovascular events in patients with CKD with 30 mL/min/1.73 m^2^ ≤ eGFR < 59.9 mL/min/1.73 m^2^ and eGFR ≥60 mL/min/1.73 m^2^ [16]. Our study corroborated this observation and expanded the patient population by including those with an eGFR between 15 and 30 mL/min/1.73 m^2^; this suggests that fibrate use may be effective in a broader range of patients with CKD. The mechanism of the beneficial impact of fibrate on MACE incidence may be explained by various effects such as reduced levels of triglycerides and total cholesterol, increased levels of high-density lipoprotein cholesterol and inhibition of albuminuria progression [16, 17]. Interestingly, a significant effect was observed for recent and current fibrate usage but not for past use, suggesting the importance of continuous fibrate therapy for cardioprotective benefits. This observation aligns with those of previous studies that highlighted the transient nature of the lipid-lowering effects of fibrates [17].

Data regarding the impact of fibrates on MACE is inconsistent among previous studies [9, 10, 17, 18]. Early studies, such as Helsinki Heart Study and Veterans Affairs and High-Density Lipoprotein Cholesterol Intervention Trial Study conducted in the 1990s, showed a reduction of MACE by fibrate alone [9, 10]. Conversely, large interventional trials conducted in the 2000s, such as the ACCORD and PROMINENT trials focusing on patients with type 2 diabetes, did not find a reduction in MACE associated with fibrate use [17, 18]. This discrepancy in the impact of fibrates on MACE could be attributed to differences in the population and study settings. Unlike the populations in the ACCORD and PROMINENT trials, our study focused on patients with CKD, not excluding those with a high risk of MACE. The objective of the present study was to investigate the association between fibrate use and the overall risk of MACE in patients with CKD. The PROMINENT study included patients with a median age of 64 years, and the ACCORD study included patients with a mean age of 62.3 years. This study used real-world data, which reflect the actual patient population, and the median age was 81 years; this may have led to different results, as many of the patients were in an age group that had at high risk of developing MACE.

Another interesting finding—a significant association of current pemafibrate use, but not bezafibrate and fenofibrate use, with the risk of MACE—suggests a possible intra-class difference of fibrates on MACE. Pemafibrate is a newer-generation fibrate compared with fenofibrate and bezafibrate. Pemafibrate, a more potent and selective activator peroxisome proliferator-activated receptor-alpha, may lead to more effective lipid-lowering effects, fewer drug–drug interactions, and fewer side effects compared with older fibrates [3, 19–21]. Furthermore, the PROMINENT study demonstrated a more pronounced decrease in serum C-reactive protein levels in pemafibrate users [18]. These anti-inflammatory effects may also contribute to a reduction in the risk of MACE. Another advantage of pemafibrate over bezafibrate and fenofibrate is the difference in metabolic pathways. While fenofibrate and bezafibrate are mainly excreted via the kidneys, pemafibrate is primarily metabolized in the liver and excreted via bile [22]. This differential metabolism may have influenced the results of our study, which focused on patients with CKD. Pemafibrate was launched in Japan in 2018, but its use remains limited. Therefore, the reported observational studies were mainly trials evaluating blood lipids, and none has investigated their association with MACE. To our knowledge, this is the first observational study of pemafibrate to explore its involvement in the development of MACE. However, because pemafibrate is contraindicated in dialysis patients in Japan, we were unable to examine its impact on the risk of MACE in dialysis patients.

The strength of this study is that patients who underwent serum creatinine testing were selected from a database covering more than 30% of the Japanese population, including those aged ≥75 years, and patients with decreased kidney functions were identified. To the best of our knowledge, no other large administrative database in Japan includes data on serum creatinine levels in elderly patients. In particular, fibrates are thought to elevate the risk of rhabdomyolysis in patients with renal dysfunction. This study is important since no intervention study using such a population has been conducted in Japan.

Some limitations of this study should be considered when interpreting the results. First, as with any observational study, causality could not be established. Second, this study used a large administrative database in Japan, which may have limited the generalizability of the findings. Third, many important confounders related to blood pressure and health status, such as the frailty index, alcohol consumption and smoking, were not accounted for, which may have affected the results. Fourth, our database has no information about urinalysis and renal imaging, which could lead to potential misclassification.

In conclusion, given the significant association between recent and current fibrate use and the reduced risk of MACE in patients with CKD, optimal use of fibrates may lead to better clinical outcomes. Moreover, among the classes of fibrates, pemafibrate may be superior to other fibrates, such as bezafibrate and fenofibrate. However, the findings should also be externally validated in countries other than Japan.

## Supplementary Material

gfad248_Supplemental_File

## Data Availability

The datasets used and/or analyzed during the current study are not open access.
