# Supplementary material for: Fibrates and the risk of cardiovascular outcomes in chronic kidney disease patients
Source: Nephrol Dial Transplant. 2023 Nov 27;39(6):1016–22. doi: 10.1093/ndt/gfad248 (PMC11139516; doi:10.1093/ndt/gfad248)
Supplement: gfad248_Supplemental_File [file gfad248_supplemental_file.docx]

**Supplemental Table**

Supplemental Table 1. Definition of comorbidities and outcomes by International Classification of Disease 10th Edition (ICD-10) codes.

| **Variable** | **ICD-10 codes** |
| --- | --- |
| ***Comorbidities*** |  |
| Diabetes mellitus | E10-E14 |
| Atrial fibrillation/flutter | I48 |
| Ischemic heart disease | I21- I25 |
| Cerebrovascular disease | G45-46, I6 |
| Peripheral vascular disease | I70, I71, I731, I738, I739, I771, I790, I792, K551, K558, K559 |
| Chronic pulmonary disease | J40-J47, J60-J67, J684, J701, J703, J841, J920, J961, J982-J983 |
| ***Outcomes*** |  |
| Major adverse cardiovascular events (MACE) | Cerebrovascular disease (G45-46, H34.1, I)  Myocardial infarction (I21, I22, I23)  Heart failure (I099, I110, I130, I132, I255, I420, I425-429, I43, I50)  Stroke (H34.1, G45, G46, I60, I61, I63, I64) |

Abbreviations: ICD-10, International Classification of Disease – 10^th^ Edition

Supplemental Table 2. Definition of medications by Anatomical Therapeutic Chemical Classification (ATC) codes.

| **Medication** | **ATC codes** |
| --- | --- |
| ACEi/ARB | C09A0, C09C0, C09D1, C9D3 |
| β-blocker | C07 |
| CCB | C08 |
| Diuretics | C03 |
| Statins | C10A1 |
| Anticoagulants warfarin | B01A0 |
| Anticoagulants DOAC | B01E0, B01F0 |
| Antiplatelet agents | B01C |
| SGLT2 | A10P |
| GLP1 | A10S |
| Glucocorticoid inhalant | R01A1, R01A3, R03F1 |
| Steroids | H02A2 |
| Nsaids | M01A |
| Opioids | N02A |
| Antidepressants | N06A |
| Fibrates | C10A2 |

Abbreviations: ACEi/ARB, angiotensin-converting–enzyme inhibitors/ angiotensin-receptor blockers; CCB, calcium-channel blockers;
